# Supplementary material for: Salmonella lipopolysaccharide stimulates uptake of long-chain fatty acids in the small intestine
Source: bioRxiv. 2026 May 20:2026.05.19.726283. Preprint. [Version 1] doi: 10.64898/2026.05.19.726283 (PMC13228628; doi:10.64898/2026.05.19.726283)
Supplement: Supplement 1 [file NIHPP2026.05.19.726283v1-supplement-1.pdf]

bioRxiv preprint doi: https://doi.org/10.64898/2026.05.19.726283; this version posted May 20, 2026. The copyright holder for this preprint (which was not certified by peer review) is the author/funder, who has granted bioRxiv a license to display the preprint in perpetuity. It is made available under a CC-BY 4.0 International license.

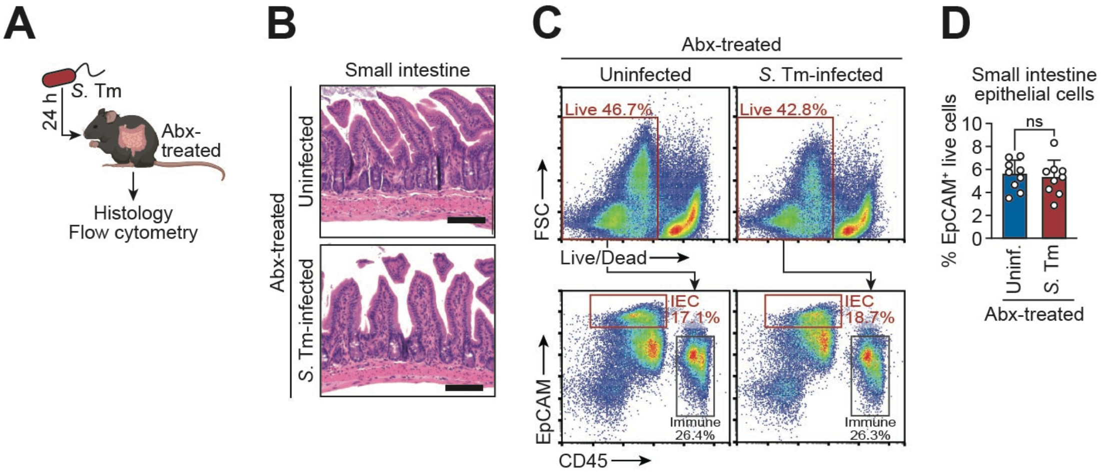

Figure S1. S. Typhimurium infection does not induce intestinal pathology or epithelial cell death at 24 hours.

(A) Experimental overview for panels B–D. Antibiotic-treated (Abx-treated) mice were orally infected with *S.* Typhimurium (*S.* Tm) for 24 hours. Small intestines were analyzed by histology and flow cytometry.

(B) Representative histological images of distal small intestines from uninfected and *S.* Typhimurium SL1344-infected mice.

(C) Representative flow cytometry plots showing gating strategy for live EpCAM$^+$ intestinal epithelial cells (IECs) from uninfected and *S.* Typhimurium SL1344 -infected mice.

(D) Quantification of live EpCAM$^+$ IECs from uninfected (Uninf.) and *S.* Typhimurium SL1344 infected mice (n=9 mice per group). Each bar graph data point represents one mouse. Significance was determined by Student's *t*-test.

bioRxiv preprint doi: https://doi.org/10.64898/2026.05.19.726283; this version posted May 20, 2026. The copyright holder for this preprint (which was not certified by peer review) is the author/funder, who has granted bioRxiv a license to display the preprint in perpetuity. It is made available under a CC-BY 4.0 International license.

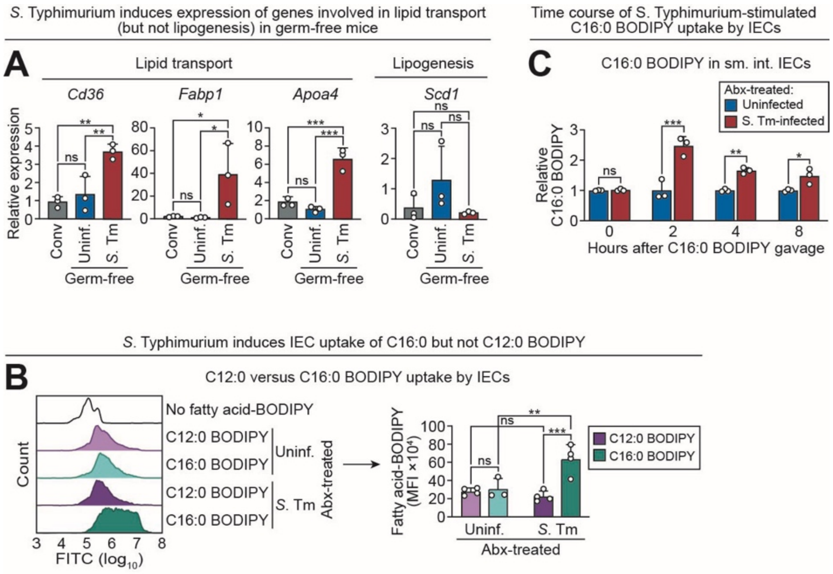

Figure S2. Supporting analyses of lipid uptake and metabolism following *S*. Typhimurium infection.

(A) qPCR analysis of genes involved in lipid transport (*Cd36*, *Fabp1*, and *Apoa4*) and lipogenesis (*Scd1*) in small intestines from conventional (Conv) mice, uninfected germ-free mice, or *S*. Typhimurium SL1344-infected germ-free mice (n=3 mice per group). Significance was determined by one-way ANOVA.

(B) IEC uptake of C12:0 BODIPY versus C16:0 BODIPY. Antibiotic-treated mice were infected and orally gavaged with BODIPY-conjugated fatty acid; IEC fluorescence was quantified by flow cytometry (n=3–4 mice per group). Representative histograms are shown on the left and quantified on the right. Significance was determined by two-way ANOVA.

(C) Antibiotic-treated mice were infected with *S*. Typhimurium SL1344 and orally gavaged with C16:0 BODIPY tracer as shown in Fig. 1I. Intestinal epithelial uptake was measured over 8 hours (n=3 mice per group per time point). Significance was determined by two-way ANOVA.

S. Tm, *Salmonella* Typhimurium; Abx-treated, antibiotic-treated; IECs, intestinal epithelial cells; Uninf., uninfected; Conv, conventional. Each bar graph data point represents one mouse.

bioRxiv preprint doi: https://doi.org/10.64898/2026.05.19.726283; this version posted May 20, 2026. The copyright holder for this preprint (which was not certified by peer review) is the author/funder, who has granted bioRxiv a license to display the preprint in perpetuity. It is made available under a CC-BY 4.0 International license.

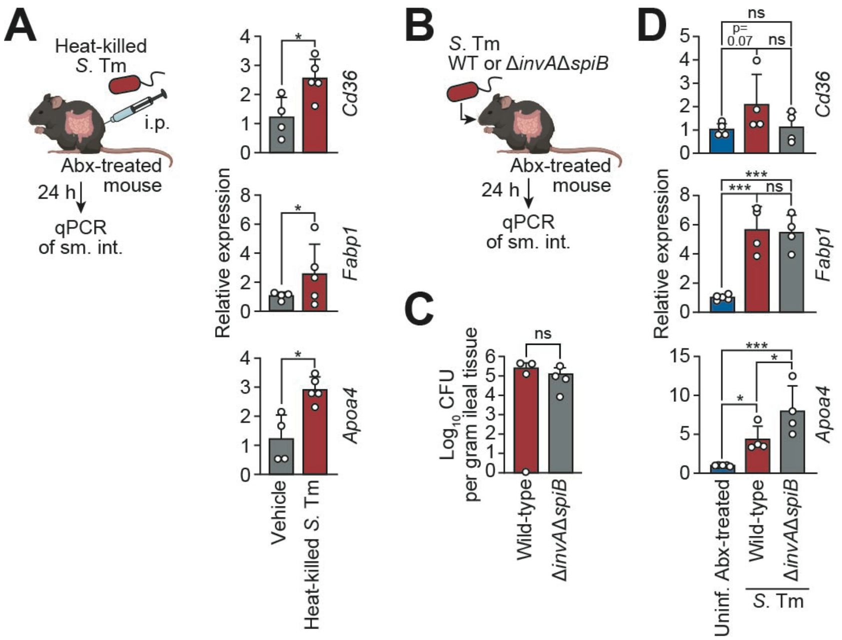

Figure S3. *S.* Typhimurium type III secretion is dispensable for lipid metabolic gene induction.

(A) qPCR analysis of lipid metabolism genes (*Cd36*, *Apoa4* and *Fabp1*) in distal small intestines from mice treated with heat-killed *S.* Typhimurium SL1344 by intraperitoneal injection. Significance was determined by Student's *t*-test.

(B) Experimental overview for panels (C) and (D). Antibiotic-treated (Abx-treated) mice were orally infected for 24 h with parental wild-type or Δ*invA*Δ*spiB* mutant *S.* Typhimurium SL1344 strains (deficient in type III secretion). Small intestines were analyzed by qPCR. Significance was determined by Student's *t*-test.

(C) Bacterial burden in distal small intestines of mice infected with wild-type or Δ*invA*Δ*spiB S.* Typhimurium SL1344 for 24 h. Significance was determined by Student's *t*-test.

(D) qPCR analysis of *Cd36*, *Apoa4*, and *Fabp1* in distal small intestines from mice infected with wild-type or Δ*invA*Δ*spiB S.* Typhimurium SL1344. Significance was determined by one-way ANOVA.

S. Tm, *Salmonella* Typhimurium; Abx-treated, antibiotic-treated; qPCR, quantitative real-time PCR; WT, wild-type; CFU, colony forming units; Uninf., uninfected. Each data point represents one mouse.

bioRxiv preprint doi: https://doi.org/10.64898/2026.05.19.726283; this version posted May 20, 2026. The copyright holder for this preprint (which was not certified by peer review) is the author/funder, who has granted bioRxiv a license to display the preprint in perpetuity. It is made available under a CC-BY 4.0 International license.

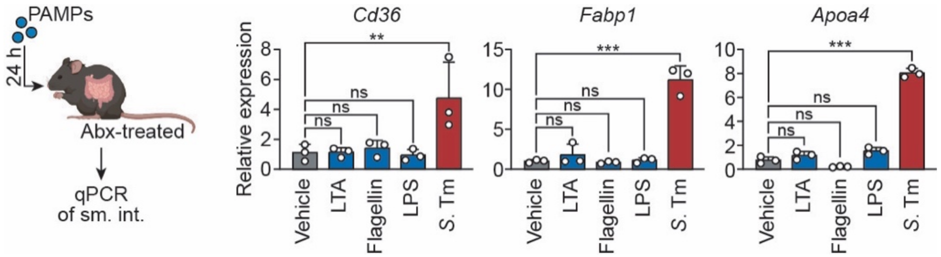

Figure S4. Oral PAMP administration fails to recapitulate infection-induced lipid metabolic gene expression. qPCR analysis of lipid metabolism genes (*Cd36*, *Apoa4* and *Fabp1*) of distal small intestines of antibiotic-treated mice gavaged with pathogen-associated molecular patterns (PAMPs): lipoteichoic acid (LTA; 1 mg), flagellin (20 µg), or ultra-pure lipopolysaccharide (LPS; 1 mg). Mice infected orally with *S*. Typhimurium as in Fig. 1D are shown for comparison. (n=3 mice per group). Significance was determined by one-way ANOVA.

Abx-treated, antibiotic-treated; qPCR, quantitative real-time PCR; sm. int., small intestine; LTA, lipoteichoic acid; LPS, lipopolysaccharide; *S*. Tm, *Salmonella* Typhimurium. Each data point represents one mouse.

bioRxiv preprint doi: https://doi.org/10.64898/2026.05.19.726283; this version posted May 20, 2026. The copyright holder for this preprint (which was not certified by peer review) is the author/funder, who has granted bioRxiv a license to display the preprint in perpetuity. It is made available under a CC-BY 4.0 International license.

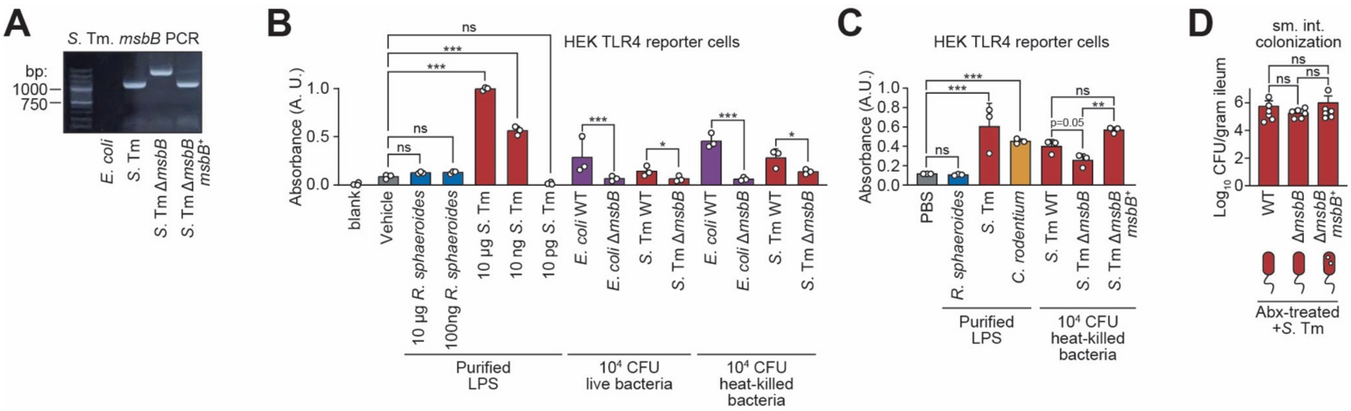

Figure S5. Characterization of *S.* Typhimurium and *E. coli* Δ*msbB* mutants.

(A) Colony PCR gel confirming mutation of *msbB* in indicated bacterial strains. Primer sequences are listed in Table S2.

(B) TLR4 activation was measured in HEK reporter cells stimulated with purified LPS from *Rhodobacter sphaeroides* or *S.* Typhimurium SL1344, or with 10^4 CFU of live or heat-killed wild-type or mutant *S.* Typhimurium IR715 or *E. coli* strains. *R. sphaeroides* expresses penta-acylated LPS that lacks the ability to stimulate TLR4 (38).

(C) TLR4 activation in HEK reporter cells stimulated with wild-type, Δ*msbB* mutant, or complemented *S.* Typhimurium IR715 strains.

(D) Bacterial burdens in distal small intestines of Abx-treated mice orally infected with wild-type, Δ*msbB* mutant, or complemented *S.* Typhimurium IR715 strains.

S. Tm, *Salmonella* Typhimurium; Abx-treated, antibiotic-treated; WT, wild-type; CFU, colony forming units; Uninf., uninfected; LPS, lipopolysaccharide; TLR4, Toll-like receptor 4; A.U., arbitrary units; sm. int., small intestine. Each data point represents one mouse. Significance was determined by one-way ANOVA.

bioRxiv preprint doi: https://doi.org/10.64898/2026.05.19.726283; this version posted May 20, 2026. The copyright holder for this preprint (which was not certified by peer review) is the author/funder, who has granted bioRxiv a license to display the preprint in perpetuity. It is made available under a CC-BY 4.0 International license.

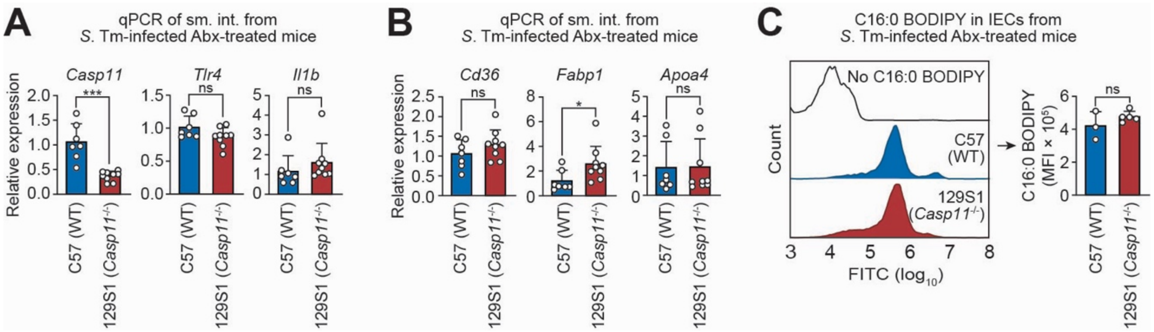

Figure S6. Epithelial lipid uptake is independent of Caspase-11–mediated inflammasome signaling.

(A) qPCR analysis of *Casp11*, *Tlr4*, and *Il1b* expression in small intestines of wild-type C57BL/6 and Caspase-11–deficient 129S1 mice 24 h after *S.* Typhimurium infection (n=7–9 mice per group). 129S1 (129S1/SvImJ) mice lack functional Caspase-11 due to a naturally occurring loss-of-function mutation in the *Casp11* gene.

(B) qPCR analysis of lipid metabolism transcripts (*Cd36*, *Fabp1*, and *Apoa4*) in small intestines of antibiotic-treated C57BL/6 and 129S1 mice 24 h after *S.* Typhimurium infection (n=7–9 mice per group).

(C) Uptake of C16:0 BODIPY by intestinal epithelial cells (IECs) from antibiotic-treated C57BL/6 and 129S1 mice 24 h after *S.* Typhimurium SL1344 infection. Fluorescence was quantified by flow cytometry; representative histograms and quantification of median fluorescence intensity are shown (n = 3–5 mice per group).

qPCR, quantitative real-time PCR; sm. int., small intestine; *S.* Tm, *Salmonella* Typhimurium; Abx-treated, antibiotic-treated; IECs, intestinal epithelial cells; Each bar graph data point represents one mouse. Significance was determined by Student's *t*-test.

bioRxiv preprint doi: https://doi.org/10.64898/2026.05.19.726283; this version posted May 20, 2026. The copyright holder for this preprint (which was not certified by peer review) is the author/funder, who has granted bioRxiv a license to display the preprint in perpetuity. It is made available under a CC-BY 4.0 International license.

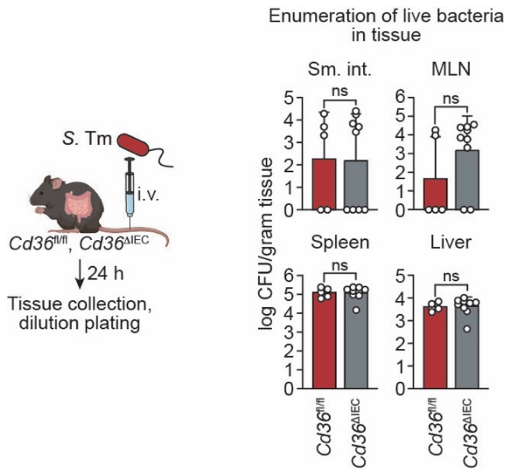

Figure S7. CD36 does not limit systemic *S.* Typhimurium burden following intravenous infection.
S. Typhimurium SL1344 burdens in the small intestine lumen, mesenteric lymph nodes, spleen and liver 24 h after intravenous infection of *Cd36*$^{\Delta IEC}$ (n=9 mice) and *Cd36*$^{fl/fl}$ littermates (n=5 mice). Data were analyzed by Student's *t*-test. *S*. Tm, *Salmonella* Typhimurium; CFU, colony forming units; sm. int., small intestine; MLN, mesenteric lymph nodes.

bioRxiv preprint doi: https://doi.org/10.64898/2026.05.19.726283; this version posted May 20, 2026. The copyright holder for this preprint (which was not certified by peer review) is the author/funder, who has granted bioRxiv a license to display the preprint in perpetuity. It is made available under a CC-BY 4.0 International license.

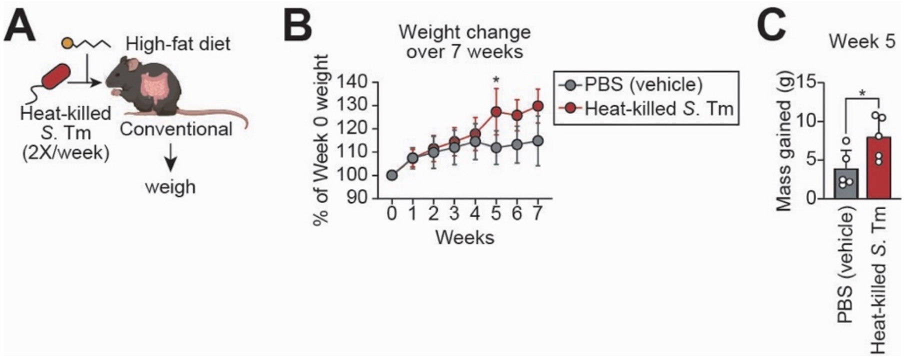

Figure S8. Intragastric treatment with heat-killed *S.* Typhimurium promotes weight gain in mice fed a high-fat diet.

(A) Conventional C57BL/6 mice were gavaged with 10^{11} CFU of heat-killed wild-type *S*. Typhimurium (*S*. Tm) SL1344 or PBS (vehicle) twice a week for seven weeks. Mice were maintained on a high-fat diet for the duration of the experiment.

(B) Weight change over the seven-week experiment. Data were first assessed for normality using the Shaprio-Wilk test. Upon confirmation of normal distribution, precent body weight change over time was analyzed by two-way ANOVA with Sidak's post-hoc test for multiple comparisons.

(C) Mass gained in *S*. Tm- or vehicle-treated mice at Week 5. Each bar graph data point represents one mouse. Significance was determined by Student's *t*-test.

bioRxiv preprint doi: https://doi.org/10.64898/2026.05.19.726283; this version posted May 20, 2026. The copyright holder for this preprint (which was not certified by peer review) is the author/funder, who has granted bioRxiv a license to display the preprint in perpetuity. It is made available under a CC-BY 4.0 International license.

Table S1: Lipid classes and abbreviations used in lipidomics analyses

Abbreviation	Lipid class
CE	Cholesterol ester
Hex2Cer	Dihexosylceramide
Cer	Ceramide
HexCer	Hexosylceramide
PC	Phosphatidylcholine
PI	Phosphatidylinositol
PS	Phosphatidylserine
TAG	Triacylglycerol
PG	Phosphatidylglycerol
LPC	Lysophosphatidylcholine
LPE	Lysophosphatidylethanolamine
DAG	Diacylglycerol
PE	Phosphatidylethanolamine

bioRxiv preprint doi: https://doi.org/10.64898/2026.05.19.726283; this version posted May 20, 2026. The copyright holder for this preprint (which was not certified by peer review) is the author/funder, who has granted bioRxiv a license to display the preprint in perpetuity. It is made available under a CC-BY 4.0 International license.

Table S2: Oligonucleotides used in this study

Description	Source	Identifier/Sequence
TaqMan *Apoa4* primer+probe	Thermo Scientific	Mm00431814_m1
TaqMan *Fabp1* primer+probe	Thermo Scientific	Mm00444340_m1
TaqMan *Cd36* primer+probe	Thermo Scientific	Mm00432403_m1
TaqMan Scd1 primer+probe	Thermo Scientific	Mm00772290_m1
TaqMan *Rn18s* primer+probe	Thermo Scientific	Mm03928990_g1
msbB cloning upstream forward	IdT	5'-ACGGCTGACGACCACACTATC
msbB cloning downstream reverse	IdT	5'-GATTTTTCGAATTCAGGGATATACTCACTATTATTTTTT TGGTTTCCATGCTTTTC
msbB cloning upstream reverse	IdT	5'-TGCCGATTTTCGCCATGTC
msbB cloning downstream forward	IdT	5'-AACGTAGCGGCGCTGCCG
pEK3 assembly *msbB* forward	IdT	5'-gcaaaagtGACAGAGCCGGTACGCGA
pEK3 assembly *msbB* reverse	IdT	5'-tgaatgggAGCACCGTCGGTTCAACC
pEK3 assembly pWSK29 forward	IdT	5'-cggtgctCCCATTCACTGCCAGAGC
pEK3 assembly pWSK29 reverse	IdT	5'-ggctctgtcACTTTTGCTTTGCCACGG
EcN *msbB* insertion mutant forward	IdT	5'-CTAGAGGTACCGCATGTTACAGTCAACGCGCGGC-3
EcN *msbB* insertion mutant reverse	IdT	5'-AGCTCGATATCGCATGCTTTCGCCACCCGCGCTA-3'
